# Medical Items and News of Interest to the Physician

**Published:** 1877-07

**Authors:** 


### For the Use of Physicians.

CULLED FROM THE STANDARD MEDICAL JOURNALS OF THE WORLD.

NOTE.—These formulæ are intended only for the regular practitioner of medicine, and should be employed only by him, or under his immediate supervision.

**Whooping Cough.**

Dr. Snowden of Camden Co., N. Y., notices his success in arresting the paroxysms of whooping cough by alum.

---

**Pneumonia.**

Pneumonia is successfully treated by Dr. Whitaker, of Cumberland Co., N. Y., by Squibb's fluid extract of ergot, 30 drop doses every three hours, fully controlling the disease in five days.

---

**Scarlatina.**

Thorough inunction of the skin by warm oil, lard or vaseline, is strongly recommended by Dr. Fisher, of Hudson, in scarlatina. It always allays irritation, reduces temperature, tends to prevent sequelæ, and diminishes the danger of contagion. *Medical Record.*

---

**Neuralgic Pill.**

℞

Quiniæ sulphatis, gr. i.

Ferri et potassæ tart gr. ij.

Morphiæ sulph gy. $\frac{1}{24}$ ad $\frac{1}{12}$. M.

Take every hour until an expected paroxysm has been missed.

---

**Ergot in Chronic Uterine Catarrh.**

As the operation of ergot given internally is very tedious, and its hypodermic injection is attended with difficulties in private practice, Dr. Goreleitschenko has tried the effect of pencilling the canal of the cervix uteri with a solution of ergot (ergotin, ℈ ij; glycerin and distilled water, of each, ʒ ij), leaving the pencil within the canal from one to three minutes. In this way he has treated twenty-nine cases of chronic metritis attended with chronic catarrh. In twenty-two of these discharge ceased, the swollen state of the mucous membrane disappearing, and the uterus itself being smaller and firmer. The subjective symptoms also ceased, and regular menstruation ensued. In the other seven cases the improvement which ensued was soon followed by relapse, the uterus in these cases having undergone more advanced alterations.—*St. Petersburg Medical Woch.*

**Acute Rheumatism.**

For the relief of acute rheumatism, salicine, 8 to 10 grains every three hours, has proved successful. For the same disease salicylic acid is much extolled.—*Medical Record.*

---

**Diphtheria.**

Dr. Miller, of Morris, N. Y., recommends proto-iodide of mercury in diphtheria, to be given until there is an increased flow of saliva, which attained, the tenacity of the membrane is less, and the patient relieved.—*Medical Record.*

---

**Remedy for Sea-Sickness.**

The *British Medical Journal*, without being able to name its authority, says that it is alleged that it has been astertained that very small doses of apomorphia, taken in water, is a cure for sea-sickness.

---

**Elixir of Pepsin, Bismuth, and Strychnia.**

℞

Acetate of Strychnia, 1 grain.

Elixir of Pepsin and Bismuth, 16 fl. ℥

N. B. The quantities of active ingredients, as iron or alkaloids, in these preparations, is supposed to be indicated or directed by the physician, who may vary them as occasion requires.

---

**Elixir of Beef, Iron and Wine.**

℞

Extract beef, (Liebig's), ½ ℥.

Ammonio-citrate of Iron, 256 grs.

Spirit of Orange. (1 : 10) ½ fl. ℥.

Distilled water, 1½ fl. ℥.

Sherry Wine, q. s. ad. 16 fl. ℥.

Dissolve the ammonio-citrate of iron in the water, dissolve the extract of beef in the sherry wine, add the spirit of orange and mix the solutions.

---

**Hydrobromic Ether as an Anæsthetic.**

Rabuteau, in a memoir read before the Academic des Sciences, states that he has investigated the physiological properties and mode of elimination of hydrobromic ether. He has satisfied himself that this anæsthetic agent, which possesses properties intermediate to those of chloroform, bromoform, and ether, might be advantageously employed to produce surgical anæsthesia. The hydrobromic ether is neither a caustic nor an irritant. It can be ingested without difficulty, and applied without danger, not only to the skin, but to the external auditory meatus and to the mucous membrane. It is eliminated completely, or almost completely, by the respiratory passages, in whatever way it may have been introduced into the system.—*Lancet.*

**Elixir of Calisaya, Iron and Bismuth.**

℞

Ammonio-citrate of Bismuth, 256 grs.
Ammonio-citrate of Iron, 256 grs.
Distilled water, warm, 2 fl. ℥.
Elixir of Calisaya, q. s. ad. 16 fl. ℥.

---

**Elixir of Pepsin and Bismuth.**

℞

Saccharated Pepsin, 256 grs.
Ammonio-citrate of Bismuth, 256 grs.
Spirit of Orange, ½ fl. ℥.
Syrup, 2 fl. ℥.
Sherry Wine, q. s. ad. 16 fl. ℥.

---

**Tonic in Anæmia.**

℞

Tinct. ferri chloridi, f. ʒ iij.

Potassii chloratis, ʒ i.

Solut. strychniæ sulph. (gr. i. ad f. ℥ i.) f. ʒ ij.

Syrupi simplicis, f. ʒ iv.

Aquæ puræ q. s. ad. f. ʒ iv.

M. Sig. Two teaspoonfuls, three times daily, as a tonic in anæmia.—DR. D. S. BRAINARD.

---

**Palpitation of the Heart.**

Dr. J. Lardies (*L' Union Med.—Clinic*) describes a method by which palpitation of the heart not due to organic lesions may be arrested at once. The patient is directed to bend the body, head down, and the arms hanging so as to momentarily cause congestion of the upper part of the body. In all cases of nervous or anæmic palpitations, the heart quickly resumes its normal functions. If respiration be arrested for a few seconds while the patient is in the above position, the relief is still more speedy.

---

**Injection of Quinine in Gonorrhœa.**

Radha Nauth Roy, Assistant Surgeon, extols (*Indian Medical Gazette*, May, 1876), the efficacy of injections of quinia in gonorrhœa. He states: "I was once tempted to try it in a case of acute gonorrhœa, where scalding was unbearable, and discharge profuse, and to my utter surprise after the third day I found the man quite relieved. He described to me the soothing effect of the injection as something cold like ice. The discharge was so much diminished that his clothes were scarcely stained after the third day. There was no more incessant desire to void the urine, and he was to all appearances comfortable. My success in this case made me bold enough to use it in other cases, and I have invariable found the disease yield both in its acute and chronic stage under its influence. It acts as a tonic and astringent to the mucous membrane of the urethra. I have also used it in some cases of cystitis with much benefit. I generally use it dissolved in sulphuric acid dil. mixed with rose water. Two grains of quinine sulph. dissolved in acid. sulph. dil. ♏ viij. or ♏ x., and mixed with an ounce of rose water—to be used twice for injection. At the same time I give copaiba mixture to my patients. In almost all the cases I have found it act like a charm. The disease is generally cured within a week, but chronic cases take a longer time. In a few acute cases it took more than a fortnight, but the delay in them was attributable to their irregular habits during this treatment."

---

**Nitric Acid for Hoarseness.**

Dr. W. Handsell Griffiths says that a few drops of nitric acid in a glass of sweetened water, a couple of times daily, will be found an excellent remedy for the hoarseness of singers. One of the largest fees ever received by him—so he says—was for this prescription.—*Southern Medical Record.*

---

**Simple Remedy to Stop Bleeding.**

Mr. F. E. Forster, of this city, informs us that lately he cut himself, and trying to stop the bleeding he did not succeed, notwithstanding he tried to do it in several ways; finally the idea struck him to put on some dry plaster of Paris, which happened to be at hand. It stopped the bleeding at once, while it only caused some stinging sensation, lasting a minute or two, but no ill effects were experienced. He requests us to publish this for the benefit of others, and we do so cheerfully, thanking him for the good example he has set by giving this communication.—*Druggists' Circular.*

---

**Prickly Heat.**

*Santiago* (*San Antonio, Texas*), writes as as follows: "Of all the many remedies employed in this troublesome affection, the most successful in my experience is a bath of cold water rendered slightly alkaline by soda, together with brisk friction with a coarse towel. The flesh brush and bathing glove help also. The following lotion, used from time to time, will give prompt relief:

Carbolic acid, ℈ ij.

Camphor, ℈ ij.

Alcohol, ℥ i.

Glycerine, ℥ i.

Water, ℥ ij.

Mix. To be used at night on retiring, not forgetting the friction.

**Ointment for Piles.**

Powdered opium, gr. xxx.
Tannin, ʒ i.
Carbolic acid, gtt. xv.
Oil of tobacco, gtt. x.
Solution of subacetate of lead, gtt. xx.
Simple ointment, ℥ i.

Mix intimately. To be used morning and night.

---

**Belladonna as a Brain Stimulant.**

In a paper read before the Maine Medical Association by Dr. T. H. Jewett, he maintained that this drug is a powerful cerebral stimulant, and is applicable to inflammation and congestion of that organ. This is only saying what the homœopaths have long proclaimed, and is the basis of a large share of their therapeutics. Who is to have the honor of the discovery? In this matter Hahnemann seems to be somewhat ahead.

---

**On the Use of Warburg's Tincture.**

Dr. Broadbent, of St. Mary's Hospital, London, claims that he has used Warburg's tincture with great success, believing, indeed, that he has saved life by it in several instances. Its efficacy he attributes to quinine, which, though present in the small proportion of nine and a half grains to the ounce, is aided in its action by powerful aromatics. To render the tincture thoroughly efficient, it should be given without dilution, at intervals of two or three hours, during which time nothing should be taken but a little beef-tea or brandy, and this only if required by the state of the patient. The applicability of the remedy is to those cases where there is no organic disease necessarily fatal and where the local inflammation is inadequate to produce the symptoms, but the nervous system is overwhelmed by a poison, as in malignant remittents, or perhaps by pyrexia simply.—*Practitioner.*

---

**Diphtheritic Sore Throat.**

Dr. Lolli, of Florence, Italy, states that he has adopted the following treatment in this affection with the best results: 1. Never cauterize the throat or abstract blood; abstain from purgatives and emetics, unless in very exceptional cases. 2. Nourish the patient according to his appetite, but let the food be light and easily assimilated. 3. Keep up the functions of the skin from the very commencement of the disease till the local, or still better, the general symptoms allow you to judge that the morbid process is extinct. (Great stress is laid on this point.) 4. For local application, as well as for internal use, the author strongly recommends the following "anti-diphtheritic mixture:"

Boiling water, oz. vj-xx.
Liquid sesquichloride of iron, m xx-dr. j.
Carbolic acid, gr. iij-xx.
Red honey, ℥ vj.

This can be used internally and as a gargle every two hours, one or two spoonfuls being a dose. This treatment gives a mortality less than two per cent.; average duration of attack, eight or ten days.—*Reporter Faliciense.*

---

**Elixir of Calisaya, Iron, Bismuth and Strychnia.**

℞

Ammonia-citrate of Bismuth, 256 grs.
Citrate of Iron and Strychnia, 128 grs.
Distilled water, warm, 2 fl. ℥.
Elixia of Calisaya, q. s. ad. 16 fl. ℥.

NOTE.—128 grains of citrate of iron and strychnia, U. S. P., contain about $1\frac{1}{4}$ grain strychnia.

---

**Treatment of Cholera Infantum.** (King.)

Leptandrin, 6 grs.

Quinia sulphate, 3 grs.

Camphor, $1\frac{1}{2}$ grs.

Ipecac, $\frac{3}{4}$ grs.

Mix and divide into 12 powders, of which one may be given every two or three hours, to be continued, if necessary, for several days.

---

**Apomorphia as an Expectorant.**

During the last two years the muriate of apomorphia has been used at the Polyclinic in Heidelberg, and is regarded as successful and reliable. Dr. Jurasy, desiring to test its power as an expectorant, gave it in minute doses in cases of tracheitis and bronchitis of the larger and smaller tubes. The results were gratifying. It liberated the firm and tenacious mucus, the sputa became copious, and the cough was much relieved. The auscultatory signs showed also that there was a decided improvement. The formula employed was: Apomorphia mur. gr. $\frac{3}{20}$ $\frac{1}{2}$; acid. muriat. gtt v.; syr. simplicis ℥ ss; aquæ ℥ iv.; a tablespoonful every two hours. It will be seen that the dose averaged between $\frac{1}{60}$ and $\frac{1}{20}$ of a gr., and this amount did not prove too large for adults. The patients stated that after the first dose they had a slightly unpleasant feeling, which did not return after subsequent doses. Apomorphia was found to have the property of becoming greener gradually, but this was overcome by the addition of a little more muriatic acid. It should be kept in black or dark colored bottles.—*Allg. Med. Central Ztg.*

**Artificial Sea-Water.**

A good article of sea-water for bathing purposes may be prepared as follows:

Common salt, 180 grains.
Chloride of calcium, 18 grains.
Sulphate of magnesia, 120 grains.
Water, 1 gallon.

---

**For Chilblains.**

Melt together in a suitable vessel, three ounces beeswax, three ounces Venice turpentine, eight ounces lard, and one pint sweet oil. Stir these well together, and raise the temperature till the mixture simmers; then allow it to cool. This should be applied to the feet on a piece of cloth when going to bed.

---

**Mosler's Tape-Worm Bolus.** (Boll tænifugi Mosler.)

℞

Florum koosso, 30 grm.
Kamalae, 15 grm.
Oleoresinae filicis maris, 4 grm.
Mellis, q. s. grm.

Mix and divide into 60 bolus, which may be sprinkled over with powdered cinnamon, and of which 30 should be taken in the evening, and 10 or 20 more the following morning.

---

**A New Anæsthetic**

Has been described by M. Rabuteau before the Academy of Sciences, Paris. It is hydrobromic ether, which, he says, can be administered without difficulty, and which is, moreover, eliminated almost completely by the respiratory passages. It holds an intermediate place between chloroform, bromoform and ether. Considering the frequent recurrence of chloroform accidents, any new anæsthetic which promises to yield a greater degree of immunity from danger of a fatal result is worthy of trial.

---

**Atropine Enemata in Cystitis.**

Dr. Simple states, in the *Virginia Medical Monthly*, that he has treated many cases of cystitis with success by administering in enemata from forty to sixty minims of a solution of sulphate of atropia (one grain to eight ounces of water), to which is added sufficient carbolic acid to prevent the formation of organic matter and the deposit of the atropia. The dose is added to half an ounce of water, and administered twice in the twenty-four hours. It uniformly and immediately arrests the frequent strangury and painful micturition, gradually checks the mucous and sanguineous discharges, and relieves the suprapubic pain with the cystic inflammation. When the urine is alkaline, Mettauer's nitro-muriatic acid is given to correct it; and when it is so acid as to irritate, the bicarbonate of potash or subnitrate of bismuth may be given, especially the latter, because of its tonic effect and its soothing influence on the mucous surfaces of the urinary organs. When constipation is present, which is frequently the case, it must be met by means of the German pulv. glycyrrhizæ co. or other aperients, until the effect of the atropia passes away, which generally occurs soon.

---

**How to Cure a Bad Cold.**

Wharton (*Virgin. Med. Monthly*) recommends carbonate of ammonia in full and often repeated doses: 10 grains, in mucilage, every hour or two hours, for one or two days in severe cases. With proper attention to other hygienic means the patient will soon recover from the severest cold without the liability to relapse which follows the employment of sweating medicines.

---

**Headache.**

Jno. E. Lockridge, M. D., in a communication to the *American Practitioner*, strongly recommends the following formula as an infallible remedy for headache:

Bromide Potassium, ℥ ij.
Tincture Aconite root, ʒ i.
Distilled water, ℥ ij.
Simple Syrup, ℥ ij.

Mix. Dose, a dessertspoonful in some water every three hours until relieved.

---

**Ammoniated Tincture of Guaiacum in Inflamed Throats.**

Dr. Garner recommended some time since (*Canada Lancet*, July, 1876) the employment of ammoniated tincture of guaiacum in inflamation of the throat whether acute or chronic. The remedy, he says, seems to be totaly unknown to some practitioners, and wholly ignored by others. He uses the remedy with almost invariable success in cases of chronic hoarseness, employing it in the form of a gargle. In the first stage of quinsy its action is astonishing. In cases of inflamed tonsils or sore throat, when produced by or accompanying measles, scarlatina, cynanche, parotiditis, and croup, he uses the pure tincture by means of a sponge-probang. His formula for a gargle is as follows:

℞

Tinct. guaiaci ammon., ʒ iij.

Liquor. potasse., ʒ iij

Tinct. opii., ʒ ij.

Aq. cinnamomi, ad. f. oz. viij.

**Iodide of Starch as an Antidote.**

Dr. Bellini, Professor of Toxicology at the Royal Institute at Florence, recommends iodide of starch as a valuable antidote in poisoning by alkaline and earthy sulphides, caustic alkalies and ammonia, and the vegetable alkalies. In poisoning by alkaline or earthy sulphides, he thinks it preferable to all other antidotes; in poisoning by caustic alkalies, it is applicable when acid drinks are not at hand.

---

**Electrolysis of Uterine Tumors.**

Dr. Cutter, of Massachusetts, devised the new grooved electrode. These pointed electrodes are driven into the most accessible part of the mass, sometimes through the abdominal walls, at other times through the vagina or rectum. The electrodes must be separated from each other by at least half an inch of tissue, more than this was better. The operation should always be performed under ether. The duration of the sitting varied from five to fifteen minutes—*Medical Record.*

---

**Treatment of Tetanus.**

The tepid bath was employed daily, sometimes for nearly an hour, and had a marked tranquilizing effect. Bromide of potassium was commenced only on the thirteenth day of the illness—which date, if cases of tetanus survived, they often recovered; but the drug seemed to be specially indicated, physiologically for the disease. Dr. Wood had recorded the results of treatment by its means in sixteen cases—nine of traumatic tetanus—all of which recovered; and seven of idiopathic tetanus, with five recoveries.

---

**Strangulated Hernia.**

The *Practitioner* states that M. d'Outrelepont, has practiced aspiration in two cases of strangulated hernia. The first case was that of a woman, aged forty-eight, who had a femoral hernia that had been strangulated for two days. The taxis had been tried without effect. A puncture was made with the needle of the aspirator, (No. 1 Dieulafoy), and about fifty grammes of sero-sanguineous liquid was withdrawn; the hernial tumor though softened could not be reduced. The symptoms calmed down and the patient left the hospital. Six months after the hernia again became strangulated, but this time taxis was applied within twenty-four hours, aud reduction was effected. The second case also occurred in a woman, aged forty-eight, affected with a crural hernea. A puncture was made with a No. 2 needle and a quantity of bloody serum removed, smelling strongly feculent. The hernia could not be reduced. The symptoms continuing, an operation was performed. On opening the sac the contents of the intestine were seen to escape from the opening made by the needle. The stricture was divided, but the intestine was not relieved; the patient died in a fortnight. D'Outrelepont concludes that in small and tense hernia, in which gangrene is apt to supervene early, aspiration ought not to be tried except during the first few hours. In large hernia, in which the progress of the malady is not so violent, aspiration may be tried at a more advanced period.—*Med. and Surg. Reporter.*

---

**To Make Leeches Bite.**

Place the leeches in a glass half full of cold water. Cleanse the part to which they are to be applied carefully with warm water, and then apply the glass containing the leeches to the part. They attach themselves with surprising rapidity. The patients often speak of the bites appearing to be simultaneous. When the animals have all become attached, allow the water to escape into a sponge or cloth, so as not to wet the patient.—*N. Y. Med. Jour., from Gazz. Med. Ital. Lomb.*

---

**Citrate of Chinoidine in Fevers.**

From the original investigations of Buchner, recently repeated by Haller at the General Hospital of Vienna, it has been ascertained that the citrate chinoidine is about as successful as the sulphate of quinine in the treatment of intermittent fever, while the cost of the former is very much less than that of the latter. Haller gives a drachm of the citrate in three ounces of water and half an ounce of cinnamon water, for two or three hours during the apyrexia. Of forty cases thus treated, only one had a return of the fever, and this one was also cured by larger doses.—*N. Y. Med. Jour., from Gazz. Med. Ital. Lomb.*

---

**Successful Treatment of Meningitis.**

In the *British Medical Journal,* Dr. J. Cross reports this case: A boy, aged six, had had persistent vomiting and headache for about a week before he was seen. The headache was paroxysmal, and so severe as to awaken the child from sleep; it was at first occipital, but afterwards frontal, and during the paroxysms the brow was very contracted. There were strabismus and dilated pupils, but they were equal and fairly sensible to light. There was also great intolerance of light and sound. The abdomen was retracted, and the *tache cerbreale* could easily be produced. The pulse was irregular and weak, and occasionally intermittent, and the respiration unequal and sighing. Improvement took place steadily under hydrargyrum cum creta in grain doses, at first every four hours, and afterward three times a day, and two grains of iodide of potassium, with three grains of the bromide, thrice daily. When convalescence began, iodide of iron and cod-liver oil were administered. A curious feature of the case was that, when he was first able to get up and move about the room, he walked with a jerky, shuffling gait and back bent almost double; but he eventually walked quite steadily and perfectly erect.

**A Pleasant Substitute for Cod-Liver Oil.**

The *Journal d'Hygiene* recommends as an agreeable substitute for cod-liver oil a soup made of fish livers and calves' or sheep's brains. This should be prepared *en puree*, as the cooks say, and may be made exceedingly palatable. The ingredients contain iodine, phosphorus, lime and fat, the active principles of the oil.

**Digestive Pastilles of Borivent.**

| | | |
|---|---|---|
| Bismuth subnitrate, | 20 | parts. |
| Calcium phosphate, | 30 | " |
| Sodium bicarbonate | 10 | " |
| Magnesium carbonate, | 20 | " |
| Iron carbonate, | 50 | " |
| Sugar, | 1000 | " |

Flavor with essence of peppermint, anise, or orange flowers. Make into pastilles of 1 gramme each, of which 3 to 12 may be taken daily.

**Injections of Cold Water in Acute Rheumatism.**

Dr. Dieulafoy (*Gaz. des. Hop.*, No. 99) has for several years past been in the habit, in acute articular rheumatism, of injecting some ten drops of cold water around different parts of the affected joint, as a means of relieving the pain. The results are most remarkable. The pains abate, and the patient is enabled to move the joint, and in some cases the rheumatism is even cured by this simple means. The same means may be employed also in muscular rheumatism, ischias, etc.

**Sunstroke in Children.**

Dr. Charles West said at a late meeting of the Royal Medical and Chirurgical Society, that sunstroke in children did not always depend on direct insolation, but might be produced by exposure to heated air, even when there was shelter from the direct rays of the sun. He had sometimes, under these circumstances, seen very general paralysis in children, as a temporary condition. He did not know of any means of deciding at the beginning of a case of paralysis in childhood, whether it was likely to be permanent, or to end in more or less complete recovery. If, however, much hyperaesthesia were present, the chance of recovery was generally less, although there were exceptions Those cases which absolutely recovered were those in which the improvement commenced early.

**Preparation of Raw Meat.**

The following directions are given by the *Jour. de Pharmacie* for the preparation of raw meat for invalids: Beef is preferable to mutton; the fat should be removed (one reason being that it may contain cysticercus). The best part is the *rump steak* (*sic*). The fibres are here best suited for rasping (*rapage*) in longitudinal direction. This is the best mode of division. Chopping removes from the meat most of its juice, and does not give such good division. The rasping is done with a sharp knife-blade—the sharper the better. The piece of meat shonld be pretty thick, and of lozenge shape; the rasping can be done on all the facings, in the natural direction of the muscular fibre. The piece should rest, held by one end, on a resistant and slightly inclined plane. The meat is generally reduced to the form of a pill or bolus, which is rolled in powdered sugar or crumbs of bread. If it cannot be taken thus it may be given under the mask of bouillon, which should be cold. One of the best methods is to prepare a thin porridge of tapioca; let it cool until it cannot cook the meat in the least. Then the meat finely rasped, is introduced into a small quantity of the cold soup till the mixture is complete. This mixture has the aspect and consistence of a fine soup of tomatoes. Next the tapioca porridge is gradually poured on this soup, the mixture being constantly stirred. Thus a homogeneous porridge is obtained, in which the meat is so well concealed that no one would detect it unless previously advised of its presence.

**Medical Uses of Myrrh.**

Myrrh, which is, perhaps, too much neglected in our time, shares the general properties of substances composed of a resin combined with an essential oil, and among these, may be placed between tar and ammoniac.

M. Deliaux de Savignac considers its action upon the stomach as being well established. He has seen painful dyspepsia rapidly ameliorated under its use. It acts as a general tonic in gastralgia dependent upon several chronic diseases, anæmia and chlorosis especially. Finally, its local calmative and disinfectant action makes it a useful addition to certain topical applications. The following formulæ are recommended, by the author referred to, in the *Dictionaire encyclopædique des sciences medicales :*

For a tooth powder :

Prepared chalk, 500 grammes.
Powdered borax, 250 grammes.
Myrrh, 125 grammes.
Orris root, 125 grammes.
M.

Antigastralgic wine of myrrh, prescribed in the dose of a wine-glassful, three times a day, before or after meals, according to the time at which the pain is experienced, has an excellent stomachic effect; it is prepared as follows :

Myrrh, powdered, ʒ v.

Bitter orange peel. ʒ iv.

Malaga wine, f ℥ xxviii.

Macerate ten days and filter.—*Jour. de Med. et de Chir. pratiques*, from *St. Louis Clinical Record.*

---

**Choice of Sedatives for the Very Young.**

Dr. Stokoe (Guy's Hospital Reports for 1876), says: "If we purpose giving a sedative to the very old or very young, we must be cautious, especially in using any of the preparations of opium, as with them they are not only prepotent, but often cumulative in their effects. As a consequence of this, for some years past I have trusted almost entirely to sedatives other than opiates, in treating children in their first septennate, and I have seen no reason to believe that any want of success has ensued from this exclusiveness. That such a precautionary measure is not altogether uncalled for, has been impressed upon me by my experience of the method of medication adopted by the more ignorant (including nurses and nursery-maids), whose frequent habit it is to increase the prescribed dose sevenfold, or to repeat it with undue persistence, if it should fall short of the expected effect; with what result may be conceived when two or three minims of laudanum have been ordered for an infant. With potassic bromide and conium for the various morbid conditions incidental to teething; chloroform for administration during the paroxysm of a convulsive attack; chloral for those derangements in which insomnia is the prevailing symptom; aconite for inflammations, fevers and feverishness generally; belladonna and hyoscyamus for many visceral disorders of a painful or obstinate nature, and combinations of these and other drugs to soothe coughs and the innumerable aches and pains of neuralgic, myalgic or rheumatic origin—to say nothing of a host of external sedative applications, many of which are very potent—we need be under no apprehension lest we should be incapable of coping with the assaults of disease in children as effectually as we could do with one more weapon in our repertory."

---

**Salicylic Acid in Diphtheria.**

Dr. Letzerich has made a number of experiments as regards the action of salicylic acid upon the organisms found in diphtherial deposits, the result showing that this acid possesses the power of killing the germs in question. He has also used salicylic acid in seven cases of the disease, five of which were mild, two severe. In the former cases a gargle according to the following formula was employed :

℞
Acid. salicylic, gr. xv.
Solve in sp. vin. rect., ♏ xxx.
Aq. destillat., ad. ℥ viii.

Under the frequent use of this gargle the diphtheritic membrane disappeared from the throat entirely in from two to four days. In the severer cases the treatment was both internal and external. Four and a half grains of the powder, with an equal quantity of sugar, were administered every two hours, and the throat was swabbed with a solution of the acid in alcohol and water (five parts acid, one part alcohol and fifty parts water.) In addition, the throat was occasionally touched with a damp camel's-hair pencil dipped in the powdered acid.

The result of this treatment was so favorable that Dr. Letzerich urges strongly its further trial. —*Druggists' Circular.*

---

**Rheumatism.**

In a recent monograph, Dr. Bremond strongly advocates the use of turpentine baths, employed by placing the patient in an apparatus where his body is enveloped in an atmosphere saturated with turpentine.

The apparatus consists of a box placed in communication with a generator of steam, and it is so contrived that at the moment when the steam enters, it is charged with turpentine, the latter being very finely divided into a kind of spray which permits of its ready absorption by the skin.

The patient is seated on an easy chair inside the box, but his head is outside, and is never exposed to the medicated vapors. The temperature of the bath does not exceed forty-five degrees centigrade, and generally forty degrees are sufficient ; and Dr. Bremond states that he has observed only twice, out of more than three thousand baths, any of that artificial exanthem which accompanies terebinthinate applications to the skin, and yet he employs for every bath more than two hundred grammes, (a gramme is about fifteen grains), of essence of turpentine, or rather of the essence of California cedar, which seems to him more efficient than that of the indigenous turpentine. The medicated steam is spread over the surface of the body by means of the apparatus and is absorbed by the skin, and Dr. Bremond considers that the turpentine thus introduced into the tissues meets the residues of nutrition when these are in excess, and by causing their combustion, prevents the undue formation of uric acid. He refers to another action of turpentine, namely, its effects on the tegumentary system, and he considers that the drug produces on the mucous membranes an effect quite as beneficial as that of its isomer copaiba.—*Med. and Surg. Reporter.*

---

**Feeding of Infants.**

In an article on this topic in the *Medical Press and Circular*, Dr. Wm. Faussett claims to establish the following leading principles:

1. That aliment should always be presented to the infant stomach in a perfectly fluid form.

2. That as bread and farinaceous substances generally have been proved by experience, and recently by numerous post-mortem examinations, to be often indigestible, and to have led directly to infant mortality, such substances had better be excluded from infant feeding.

3. That cow's or goat's milk, when pure and modified as much as possible to resemble human milk, will often be found sufficient without any other help, to nourish the new-born infant.

4. That as cocoa contains all the elements indispensible for the growth and development of the body, and can always be presented in a fluid form, it is, next to milk, preferable to all other natural substances as an article for infant aliment.

There is one other point which, though indirectly connected with infant feeding, is one of paramount importance as regards the present and future health of the individual, viz.: the necessity of guarding against the hateful practice of covering the child's face as it sleeps.

The mistaken kindness and over-zealous attention of nurses in excluding the pure air of heaven from entering the lungs, in order to guard against the effects of cold, will often be exhibited in the soft, pale, flabby condition of the infant's body, while a cachectic condition of the blood will be insidiously germinated, which must prevent the infant thriving for the present, and possibly may lay the foundation of tubercular and other diseases in after life.—*Med. and Surg. Reporter.*

---

**Curative Effects of mild continued Counter-irritation of the Back in cases of General Nervous Debility and Spinal Irritation.**

Dr. Arthur Gamgee recommends the compound mustard liniment, (Br. Ph.) as the best available counter-irritant in these cases, as it produces a remarkably active irritation of the sensory nerves of the skin, which subsides to a great extent when the preparation is removed, but which can be renewed almost indefinitely without leading to any eczematous, pustular, or ulcerative condition. He finds this plan of treatment more successful than the use of iron, cod-liver oil, phosphorus, or the constant galvanic current. The theory of its action which he gives is, that counter-irritation exerts a tonic action on the local vasomotor nerve centres.

In consequence of the expensive character of the essential oil of mustard, and the ethereal extract of mezereon which enter into the composition of this liniment, it is very commonly adulterated and is nearly inactive. When properly prepared, the liniment should possess a very pungent odor, and should produce an almost painfully acute sensation in the nostrils when it is smelled. If properly prepared a few drops of linimentum sinapis sprinkled over a pad of cotton-wool ten or twelve inches long and four or five inches broad, will suffice to produce, in a few minutes, pretty intense redness of the skin of the back, accompanied by more or less of the painful or burning sensation characteristic of mustard.

The general result of the use of this mode of counter-irritation is thus described: "On the first or second day of the treatment, the patient, if a delicate hysterical girl, may complain that the pain caused by the mustard is almost unbearable, and she may declare that the application cannot be continued. By diminishing the amount of mustard oil used, however, all such urgent objections on the part of the patient are removed. As soon as the application has been so controlled as to bring on merely an active glow and not unpleasant tingling of the skin, the patient declares that the increase in her strength is marvellous; the pain in the back and limbs undergoes a diminution, or, as long as the mustard counter-irritation is kept up, are completely in abeyance, the irritability of temper diminishes, and simultaneously the general health undergoes marked improvement.

"The increased feeling of vigor produced by the treatment is not illusory; as a rule, I have found that the improvement thus commenced has kept up so that a hysterical girl, who has been for some weeks confined during the day to a couch, to which she could with difficulty make her way from her bedroom, has in a few days cheerfully taken walks of considerable length."—*The Practitioner.*

---

**Chloral Hydrate for Burns.**

In the Philadelphia *Medical and Surgical Reporter*, Dr. S. S. Riddell, of Chippewa Falls (Wis.), relates the case of a bad scald in the face, and his experience with the above mentioned preparation. His formula was—

Hydrate of Chloral, ʒ iij.

Carron oil, ℥ vj.

This was applied by means of cotton wool, the face being completely covered with it. Within ten minutes after the arrival of the doctor at the house all pain had ceased, and within twenty minutes the patient was asleep. This is the only instance reported, so far as we know, of its use for a similar purpose. The effect seems satisfactory, especially as the patient within a few days was quite well, although the local injury to the skin and one eye had been very severe. What, it may be asked, had the carron oil to do towards allaying pain and preventing inflammation? This oil is a preparation which had long been highly esteemed in the treatment of burns. Who knows, in fact, what good the chloral did, if it did any? We are here inclined to add some testimony of our own. A couple of months ago a servant maid in our house was accidentally scalded by the cook with boiling fat. The injury extended from the elbow to the wrist on the outside of the arm. Three or four hours afterwards she showed it to us for advice. It was giving great pain, as such burns always do, and there was a good deal of redness and some little swelling, though only here and there vesication. We happened to have among our miscellaneous lot of medicines a bottle of *camphor-chloral*—a liquid caused by the interaction of these two substances broken into small bits, and placed in the bottle together. The arm was first smeared in a gentle manner with this article until it was thoroughly covered, and then a piece of soft, thin manilla paper was wrapped carefully about the suffering part. The pain abated directly, and the girl went off about her usual business. She was not, it is presumed, inclined to sleep. The next day it was dressed again by mixing camphor-chloral with a greasy base, to render it more easy of application; simple ointment of oxide of zinc was used for the purpose. This was spread over a soft rag, and covered with the manilla paper as before. We never saw the arm afterwards. All suffering was ended, and in a few days she herself had pronounced the case cured and had removed the wrappings.

---

**Bacteria, and What will Kill Them.**

In *The Circular* of January last was an article upon *Bacteria* by Prof. Tyndall, which presented a remarkable view of that microscopic vegetable in the production of the phenomena of fermentation. Below we present a short communication that appeared recently in the New York *Times*, which shows their wonderful tenacity of life, and what will not and may destroy their vitality.

One of the most active and dangerous forms of bacteria, the micrococcus, is about the shape of the head of a small pin; or, rather, when magnified 800 times, it looks like this: o o o o o. Another, the true bacteria, or rod-like particles, are about the following size and shape: ⬭⬭⬭.

But the principal point is to find out what substances or medicines will destroy them. Quinine will not, for bacteria will live and flourish on a solution of twenty grains to two drachms of fluid. Nor will camphor, for they live on a solution of thirty grains to two teaspoonfuls of fluid. For five days they were seen swimming about among the pieces of camphor, and increasing immensely in numbers. Ten drops of carbolic acid in two drachms of fluid will not kill them. They also flourish in a solution of tar, and will swim about for six or more days between particles of ten grains of calomel in two teaspoonfuls of fluid. One drachm of laudanum in two teaspoonfuls of fluid filled with bacteria will only commence to benumb and kill them at the end of six days.

They lived for ten days in a solution of tincture of nux vomica in two drachms of bacteria fluid. Tannin is the first remedy which has a decidedly destroying effect upon them. It will kill them in two hours; and although they will come to life again after being frozen in ice, and boiled in hot water, yet they will not do this after tannin is applied. Chloroform seems to kill them, yet they will come to life again. They will live in a solution of one drachm of chloral to two of water. A concentrated solution of copperas, or sulphate of iron, will kill them; also chlorine water, and dilute muriatic, sulphuric, and nitric acids.

We may draw the inferences that quinine, calomel, and carbolic acid are useless in diphtheria. That opium, nux vomica, chloroform, and chloral are comparatively so; and that tannin, sulphate of iron, chlorate of potash, chlorine water, and the dilute mineral acids may prove the only really useful remedies.

---

**Diphtheria: What is the Best Remedy?**

This most serious and, as it seems, hitherto scarcely curable disease by remedial means, if we judge from the frightful mortality that often distinguishes its prevalence, is almost certainly arrested, according to reports, by the use of *sulpho-carbolate of soda.* Two different writers in late numbers of the *Medical and Surgical Reporter* speak very strongly in favor of its signal efficacy. Dr. Anthony, of Providence, says that in fourteen cases he has given it with satisfactory effect in every instance. He administers from the beginning, every one, two or three hours, from three to ten grains, according to the condition of the patient. The doctor says: "I combine it in some cases with tinct. ferri mur., or sulph. quiniæ. It may be given in any aromatic water or syrup, but I prefer the syr. aurantii cort. In several cases where it was used it seemed to exert almost a specific effect. Topical applications were also used, of which I prefer the argenti nitras, applied either in a strong solution by means of a probang, or the solid stick applied direct to the parts.

"The following is a typical case, illustrating my course of treatment:

"S. G. T., aged five years, was first seen by me on Monday, November 13th. His condition was then as follows: Pulse, 140; temperature, 104 deg.; skin, dry; tongue, coated with heavy white fur in the centre, with a bright red edge; deglutition was performed with difficulty; the fauces were swollen, with diphtheritic patches on both tonsils. I ordered—

℞

Sulphocarb. sodii, ʒ ij.

Quiniæ sulph., ʒ j.

Acid. sulph. aromat., ʒ j.

Syr. aurantii cort., ad. ℥ iv.

M. A teaspoonful to be given every three hours.

"The throat to be showered by means of a syringe, once an hour, with a mixture of chlor. potass., mur. tinct. ferri and water.

"On Thursday my patient was up and able to eat dinner with the family."

The "question arises," what was the use of the quinine and the other means employed? Therefore, how much credit is to be given to the soda?

Dr. Greenamyer, of Smithville, Ohio, sends the following reinforcing communication, dated Dec. 12th, 1876:

"I notice in the *Reporter*, November 25th, 1876, an article from Dr. W. E. Anthony, of Providence, Rhode Island, on the use of sulpho-carbolate of soda in the treatment of diphtheria. I have had a similar experience with the use of this drug in the disease mentioned. I read a report of a case partly thus treated before the Union Medical society of Northeastern Ohio, in May last, and also the memoranda of other cases, in which I used the sulpho-carbolate from the beginning of the complaint. I attribute great value to its use, having exhibited it with marked success in about twenty cases, and I may justly say that I have never used it without good results. With sulpho-carbolate of soda, glycerine and dilute carbolic acid, whisky and milk, and warm inhalations, I have cured cases of diphtheria which, judging from their severity, would have proved certainly fatal under the old plan of treatment. I think it a remedy which deserves investigation."

Sulpho-carbolate of soda, it may be admitted, is a valuable remedy in diphtheritic affections, but we find in an "exchange" that Dr. Chenery, of Boston, has discovered *hyposulphite of soda* to be a specific. The dose is from five to fifteen grains or more, and may be administered in syrup every two or three hours, according to age and other circumstances. If too much be given it will purge, but nothing more. As much as the patient can take without affecting the bowels may be considered in some cases the proper quantity to give. The amount for thorough stimulation is greater than can be taken in water. Doctor Chenery usually gives it in such doses as can be easily taken in milk, using milk besides as food for small children. But, as milk remains undigested in the presence of hyposulphite of soda, it should not be taken as diet until an hour or so has elapsed after giving the medicine.

Dr. C. closes with saying: "Judging in this disease as I judge in others, I am fully persuaded that the treatment I have so long used, and which has not failed me yet [in 158 cases], will save nearly every case of diphtheria if seasonably and vigorously employed, and there is no reason why it should not do as well in the hands of others as in my own. In none of my cases have I used any alcohol."

This abnegation of alcohol rather conflicts with the milk and whisky diet of Dr. Greenamyer, who is quoted above.

